# Insights from an N3C RECOVER EHR-based cohort study characterizing SARS-CoV-2 reinfections and Long COVID

**DOI:** 10.1038/s43856-024-00539-2

**Published:** 2024-07-11

**Authors:** Emily Hadley, Yun Jae Yoo, Saaya Patel, Andrea Zhou, Bryan Laraway, Rachel Wong, Alexander Preiss, Rob Chew, Hannah Davis, M. Daniel Brannock, Christopher G. Chute, Emily R. Pfaff, Johanna Loomba, Melissa Haendel, Elaine Hill, N3C and RECOVER consortia, Richard Moffitt

**Affiliations:** 1 RTI International, Durham, NC USA; 2 Emory University, Atlanta, GA USA; 3 Stony Brook University, Stony Brook, NY USA; 4 University of Virginia, Charlottesville, VA USA; 5 University of North Carolina, Chapel Hill, NC USA; 6 Patient Led Research Collaborative (PLRC), Calabasas, CA USA; 7 Johns Hopkins University, Baltimore, MD USA; 8 University of Rochester Medical Center, Rochester, NY USA

**Keywords:** Epidemiology, Biomarkers

## Abstract

**Background:**

Although the COVID-19 pandemic has persisted for over 3 years, reinfections with SARS-CoV-2 are not well understood. We aim to characterize reinfection, understand development of Long COVID after reinfection, and compare severity of reinfection with initial infection.

**Methods:**

We use an electronic health record study cohort of over 3 million patients from the National COVID Cohort Collaborative as part of the NIH Researching COVID to Enhance Recovery Initiative. We calculate summary statistics, effect sizes, and Kaplan–Meier curves to better understand COVID-19 reinfections.

**Results:**

Here we validate previous findings of reinfection incidence (6.9%), the occurrence of most reinfections during the Omicron epoch, and evidence of multiple reinfections. We present findings that the proportion of Long COVID diagnoses is higher following initial infection than reinfection for infections in the same epoch. We report lower albumin levels leading up to reinfection and a statistically significant association of severity between initial infection and reinfection (chi-squared value: 25,697, *p*-value: <0.0001) with a medium effect size (Cramer’s *V*: 0.20, DoF = 3). Individuals who experienced severe initial and first reinfection were older in age and at a higher mortality risk than those who had mild initial infection and reinfection.

**Conclusions:**

In a large patient cohort, we find that the severity of reinfection appears to be associated with the severity of initial infection and that Long COVID diagnoses appear to occur more often following initial infection than reinfection in the same epoch. Future research may build on these findings to better understand COVID-19 reinfections.

## Plain language summary

More than three years after the start of the COVID-19 pandemic, individuals are frequently reporting multiple COVID-19 infections. However, these reinfections remain poorly understood. Here, we investigate COVID-19 reinfections in a large electronic health record cohort of over 3 million patients. We use data summary techniques and statistical tests to characterize reinfections and their relationships with disease severity, biomarkers, and Long COVID. We find that individuals with severe initial infection are more likely to experience severe reinfection, that some protein levels are lower, leading to reinfection, and that a lower proportion of individuals are diagnosed with Long COVID following reinfection than initial infection. Our work highlights the prevalence and impact of reinfections and suggests the need for further research.

## Abstract (web summary)

Hadley et al. characterize COVID-19 re-infections utilizing electronic health record study cohort data of over 3 million patients. They find severe initial COVID-19 infection linked to severe reinfections and less frequent long COVID diagnosis after reinfection.

## Introduction

Throughout the COVID-19 pandemic, hundreds of millions of SARS-CoV-2 cases have been confirmed worldwide^1^. However, an infection with SARS-CoV-2 does not confer lasting immunity, particularly in the context of immunologic escape displayed by new variants^2^. Reports of SARS-CoV-2 reinfection are well documented, and whole genome sequencing analysis has confirmed reinfections from SARS-CoV-2 variants that are genetically distinct from an initial SARS-CoV-2 infection^3^. Reinfections are concerning because they may interfere with the development of herd immunity^4^.

Incidence estimates of documented reinfections among persons who experienced a SARS-CoV-2 infection are low, ranging from 0.2% to 5.5%^5–8^. A review of laboratory studies found that the time from primary SARS-CoV-2 infection to reinfection can range from 19 to 293 days^9^. Data from 18 jurisdictions collected between September 5, 2021 and December 31, 2022, found that the median interval between infections ranged from 269 to 411 days^10^. Guidelines generally suggest that a new positive COVID-19 antigen or polymerase chain reaction (PCR) test should be considered a reinfection if it occurred at least 60–90 days after initial infection^5,6,8,11–14^. A few studies document cases of two or three infections, noting that third infections were mainly associated with the transmission of the Omicron variant^13,14^.

Biomarkers are an important tool for characterizing a disease. Existing research has explored the relationship of the severity of COVID-19 with biomarkers such as laboratory indicators of inflammation, dysregulated coagulation, and end-organ dysfunction^15–17^. A systematic literature review found significant associations between specific biomarkers and Long COVID symptoms^18^. Although studies of reinfection have been less common, one study that characterized patients with suspected reinfection showed increased rates of metabolic failure and similar rates of renal and hepatic failure with reinfection compared to their index encounter but did not further analyze these findings using laboratory biomarkers^16^. Most studies of biomarkers related to COVID-19 infection are limited to the time period during infection, with limited insight into the trajectories of laboratory measurements as predictors of reinfection.

Considerable interest exists regarding the severity of reinfection as compared to initial infection. Hospitalization can be an indicator of disease severity because more severe disease often requires treatment. Studies looking at reinfection and hospitalization have generally found that rates of hospitalization following reinfection were similar to or lower than rates of hospitalization following initial SARS-CoV-2 infection^19–21^. One study found that the reduced risk of hospitalization following reinfection persisted when disaggregated by age^21^. No study, to the best of our knowledge has clearly disaggregated by severity of hospitalization, such as considering the distinction between an emergency department (ED) visit, an inpatient hospitalization, and an inpatient hospitalization requiring intensive care.

Less attention has been given to the relationship of reinfections to post-acute sequelae of SARS-CoV-2 infection (PASC) or Long COVID^22^. PASC is understood as complications resulting from SARS-CoV-2 that persist or occur de novo for at least 4 weeks post-infection, and Long COVID is the clinical diagnosis for these conditions. Long COVID is associated with commonly reported symptoms, including fatigue that interferes with daily life, fever, cough, sleep problems, difficulty breathing, and difficulty thinking^23^. Existing work suggests that reinfection can increase the risk of post-acute sequelae in the pulmonary and broad array of extrapulmonary organ systems^24,25^. Additional knowledge about the relationship between reinfections and Long COVID could help inform interested parties who may be concerned that reinfections could contribute to the incidence of Long COVID.

We seek to contribute to the growing literature on SARS-CoV-2 reinfections with findings from a large cohort of more than 3 million individuals in the electronic health record (EHR)-based N3C Data Enclave. We first characterize reinfection by describing incidence and attributes. We validate findings from other reinfection studies related to reinfection incidence, the occurrence of most reinfections during the Omicron epoch, and evidence of multiple reinfections with analyses from this larger cohort. We then consider biomarkers captured in EHR data between the index date and reinfection and report lower albumin levels leading up to reinfection. We explore the severity of reinfection as measured by hospitalization and find a statistically significant association of severity between initial infection and reinfection (chi-squared value: 25,697, *p*-value: <0.0001) with a medium effect size (Cramer’s *V*: 0.20, DoF = 3). We share that individuals who experienced severe initial and first reinfection were older in age and at a higher mortality risk than those who had mild initial infection and reinfection. We assess differences in incidence rates of Long COVID following initial infection and reinfection and report that the proportion of Long COVID diagnoses is higher following initial infection than reinfection for infections in the same epoch. Finally, we discuss findings and suggest opportunities for further research.

## Methods

This study uses individual EHR data stored in the N3C Data Enclave as part of the NIH Researching COVID to Enhance Recovery (RECOVER) Initiative. The RECOVER Initiative seeks to understand, treat, and prevent PASC. For more information on RECOVER, visit https://recovercovid.org. The N3C Data Enclave provides access to harmonized EHRs from more than 75 health sites with data from over 16 million patients^26,27^. We used N3C data from version 141 (9/14/2023), which has 68 contributing sites, for the current investigation. The N3C Data Enclave’s Palantir Foundry platform (2021, Denver, CO), a secure analytics platform, was used for data access and analysis.

### Institutional Review Board

The N3C data transfer is performed under a Johns Hopkins University Reliance Protocol # IRB00249128 or individual site agreements with NIH. The N3C Data Enclave is managed under the authority of the NIH; information can be found at https://ncats.nih.gov/n3c/resources. The N3C received a waiver of consent from the NIH Institutional Review Board under the 1996 Health Insurance Portability and Accountability Act privacy regulations for a Limited Data Set.

### Key definitions

We describe the following key definitions for the study cohort, reinfection, COVID-19 variant epochs, and Long COVID.

#### Study Cohort definition, inclusion, and exclusion criteria

The study inclusion criteria include (1) having an International Classification of Diseases-10-Clinical Modification (ICD-10) COVID-19 diagnosis code (U07.1) or a positive SARS-CoV-2 PCR or antigen test between March 1, 2020, and December 31, 2022; the earliest of these events was considered the COVID-19 index date; (2) reinfection events (if any) occurring before March 1, 2023; (3) being 18 years of age or older; (4) having at least two recorded healthcare visit in the year prior to index; (5) having at least one recorded healthcare visit more than 60 days after the COVID-19 index date; (6) being from a hospital partner with data that has been updated in the last three months prior to March 1, 2023; (7) being from a hospital partner with at minimum 100 hospitalizations related to a first known COVID-19 infection and at minimum 25 hospitalizations related to a COVID-19 reinfection. A total of 3,104,391 individuals met these criteria.

#### Definition of reinfection

A COVID-19 reinfection was defined as a positive SARS-CoV-2 PCR or antigen test that occurred 60 or more days after a COVID-19 infection index date. The date of the test was considered the first COVID-19 reinfection index date. Subsequent reinfections were defined as a new positive SARS-CoV-2 PCR or antigen test that occurred 60 or more days after each reinfection index date. Although a threshold of 90 days for reinfection post-index date is common in the literature, other findings suggest that nearly all patients stop shedding SARS-CoV-2 within 60 days of infection, and many stop shedding much sooner than that^28–31^. Based on these findings and support from the RECOVER clinician advisory panel, 60 days was selected as a more appropriate threshold.

#### Definition of the COVID-19 variant epoch

We define the following COVID-19 variant epochs based on the patient’s COVID-19 diagnosis code (U07.1) or a positive SARS-CoV-2 PCR or antigen test date: Ancestral COVID-19 (March 01, 2020–September 30, 2020), Alpha/Beta/Gamma variant (October 1, 2020–May 31, 2021), Delta variant (June 1, 2021–November 30, 2021), Omicron BA.1 & BA.2 variant (December 1, 2021–April 30, 2022), Omiciron BA 2.12 (May 1, 2022–November 30, 2022) and Omicron BQ.XBB variant (December 1, 2022–March, 2023)^32^.

#### Definition of severity of COVID infection

Severity of COVID infection was identified by records of COVID-associated hospitalization, which was defined as an inpatient visit with a start date 1 day prior to 16 days after the COVID-19 index date with a COVID ICD-10 diagnosis code used during the visit. A COVID-associated ED visit was defined as an ED visit with a start date 1 day prior to 16 days after the COVID-19 index date and with a COVID ICD-10 diagnosis code used during the visit. These thresholds were intended to capture hospitalizations and ED visits that are related to COVID. Severity of infection is assessed by applying these hospitalization and ED visit criteria windows. Four levels of severity are considered: mild infection that does not temporally align with an ED visit or hospitalization, mild infection that aligns with an ED visit, moderate infection that aligns with a hospitalization, and severe infection that aligns with hospitalization and use of ECMO, IMV, or vasopressors. Vasopressors were included in addition to the more intensive ECMO and IMV because some hospitals may be limited in their ability to provide ECMO or IMV.

#### Definition of long COVID

Patients with a Long COVID diagnosis were identified with the U09.9 or B94.8 ICD-10-CM diagnosis codes. The U09.9 code was implemented in October 2021 for providers to use in a clinical setting with patients experiencing ongoing conditions after a COVID-19 infection, commonly understood as Long COVID. Many hospital sites appear to have rapidly adopted the use of U09.9 once it became available^33^. The B94.8 code is not specific to COVID-19 and instead represents sequelae of other specified infectious and parasitic diseases. This code was rarely used prior to the pandemic, but it started seeing considerably more use in November 2020. The use of this code is understood to represent Long COVID diagnoses prior to the availability of U09.9^33^. For the purposes of the Long COVID analysis, we limited the study cohort to individuals at sites that had at least 250 uses of either the U09.9 code after October 1, 2021, or the B94.8 code after November 1, 2020. Eligible reinfections for U09.9 or B94.8 had to occur after the respective dates of use of the codes. This subcohort included 1,568,810 individuals.

### Statistical analysis

In this work, we perform three main analyses focused on characterizing reinfection, understanding reinfection severity, and exploring the relationship between reinfection and long COVID. Differences in median biomarker levels were analyzed using the Wilcoxon Rank Sum Test. Tests were two-sided and -*P*-values <0.05 were considered significant. All analysis and visualization were done in the N3C Enclave using SQL, Python (v3.6), and R (v3.6), including ggplot2, survival, and survminer packages.

#### Characterization of reinfection

We used two approaches to characterize reinfection. The first is a cohort summary where we calculate summary statistics related to reinfection and disaggregate by demographic characteristics. Age, sex, race, and ethnicity were disaggregated by categories available in the N3C Enclave. We assessed comorbidities with the Charlson Comorbidity Index (CCI), a score used to assess the risk of mortality where higher scores signify greater risk and more complex comorbidities^34,35^. Vaccination rates for disaggregation by vaccination status were expected to be low because there is no explicit indicator of non-vaccination in the N3C data Enclave and vaccine reporting is inconsistent by site. In addition, the analysis timeframe includes periods when vaccines were not available. Chi-square tests were used for categorical variables, and Student’s *t*-test or ANOVA was used for continuous variables (Table 1, Supplementary Data 2, 3). Tests were two-sided. Time to reinfection analysis was based on Kaplan–Meier curves from the *survival* package in R. This analysis was performed using the date of the COVID-19 index date (the date of earliest diagnosis or positive test) and the date of the event (first reinfection date) as endpoints. Effect sizes were calculated with Cohen’s *D* or Cramer’s *V*.

**Table 1:** Descriptive characteristics of reinfected and non-reinfected COVID-19-positive patients

Category	Variable	No reinfection (*N* = 2,891,407)	Reinfected patients (*N* = 212,984)	Total (*N* = 3,104,391)	*P* value	Cramer’s *V*
Age, mean (SD)	Age	49.95 (18.7)	45.77 (18.0)	49.66 (18.5)	<0.0001	0.227 (Cohen’s *D*)
Sex (*N*, %)	Female	1,777,007 (61.5)	141,432 (66.4)	1,918,439 (61.8)		
	Male	1,113,576 (38.5)	71,477 (33.6)	1,185,053 (38.2)	<0.0001	0.026
	No sex Information	824 (0.03)	75 (0.04)	899 (0.03)		
Race (*N*, %)	White	2,148,414 (74.3)	158,389 (74.5)	2,306,803 (74.3)		
	Black	360,096 (12.5)	31,215 (14.7)	391,311 (12.7)		
	Asian	74,961 (2.6)	3313 (1.6)	78,274 (2.5)		
	Others	51,292 (1.8)	4711 (2.2)	56,003 (1.8)	<0.0001	0.025
	No race Information	256,644 (8.88)	15,356 (7.21)	272,000 (8.76)		
Ethnicity (*N*, %)	Not Hispanic	2,447,243 (84.64)	181,555 (85.24)	2,628,798 (84.68)		
	Hispanic	233,933 (8.09)	20,877 (9.8)	254,810 (8.2)	<0.0001	0.014
	No ethnicity information	210,231 (7.3)	10,552 (4.9)	220,783 (7.1)		
Charlson comorbidity index score (*N*, %)	0	1,559,754 (53.9)	113,109 (53.1)	1,672,863 (53.9)		
	1–3	856,218 (29.6)	60,179 (28.3)	916,397 (29.6)		
	≤4	299,753 (10.4)	24,524 (11.5)	324,277 (10.5)	<0.0001	0.0156
	Not available	175,682 (6.08)	15,172 (7.12)	190,854 (6.15)		
Vaccination (*N*, %)	Vaccinated prior to COVID index	723,677 (25.03)	30,340 (14.25)	754,017 (24.29)	<0.0001	0.0636

The second approach to characterizing reinfection is with biomarkers. We explored the trajectories of various biomarkers around COVID-19 initial and subsequent index dates from patients with and without reinfection. Biomarker measurements included laboratory values of ferritin, fibrinogen, C-reactive protein, procalcitonin, white blood cell count, absolute lymphocyte count, absolute neutrophil count, erythrocyte sedimentation rate, albumin, D-dimer, alanine transaminase (ALT), aspartate transaminase (AST), and serum creatinine. Units were harmonized across data partners, and clinically infeasible values were excluded according to standard N3C data quality protocols^27^. Measurements were taken from 100 days prior to and 180 days after the COVID-19 index date in patients with and without reinfection. The same time frame was used for collection around the first reinfection index date in individuals with at least one reinfection. Laboratory values were reported separately for hospitalized and nonhospitalized patients. The median laboratory value of each biomarker, with upper (75%) and lower (25%) quartiles, was binned by 7-day intervals and visualized according to time from COVID-19 infection or reinfection index date. For patients with more than one measurement of the same laboratory test in a day, values were averaged.

#### Analysis of severity of reinfection

We compared the severity of the first COVID-19 infection versus the severity of the first reinfection using a pivot table with selected row, column, and table percentages along with a chi-square test for association. Cramer’s *V* is used to assess effect size for the chi-square test. Death after initial infection or reinfection is also included in the table.

#### Reinfections and Long COVID

The subcohort of individuals described in the section “Study Cohort definition, inclusion, and exclusion criteria” was used for the analysis of reinfections and Long COVID. Kaplan–Meier curves were calculated to explore the differences in time to Long COVID diagnosis following initial infection versus reinfection. Time-to-event analysis was performed using the initial COVID-19 index date and with the first reinfection index date (for those with one or more reinfections) to the first B94.8 or U09.9 diagnosis.

### Reporting summary

Further information on research design is available in the Nature Portfolio Reporting Summary linked to this article.

## Results

The study cohort included 3,104,391 adults (age: mean 49.7 years, standard deviation (SD) 18.5; 62.8% female) (Table 1, Supplementary Data 1). The study cohort contains data from 54 health facility data partners. 73% of the cohort were included based on a documented positive PCR or antigen test alone or in combination with a COVID-19 ICD-10 diagnosis code as the COVID index date. The remaining 27% were included based on usage of a COVID-19 ICD-10 diagnosis code with no documented PCR or antigen test in the following 7 days (see Supplementary Fig. 2). These individuals with only a COVID-19 ICD-10 diagnosis code used for COVID index date were older (*p* < 0.0001) and had more comorbid conditions (*p* < 0.001) than those with only a PCR or antigen test for COVID index date (Supplementary Data 4).

Women make up more than three-fifths of the study cohort and a larger proportion of individuals with reinfections. The skew in sex can be attributed to the inclusion requirement for at least two visits in the year prior to COVID diagnosis; the male/female sex ratio is 0.80 in the initial cohort, and 0.67 after the visit requirement is introduced (Supplementary Fig. 1). The sex ratio of individuals hospitalized with COVID-19 was more balanced (0.85) (Supplementary Data 2).

### Characterization of reinfection

Table 1 describes the study cohort and highlights differences between the subgroup with no reinfections and the subgroup with at least one reinfection. A total of 6.9% of the study cohort had at least one documented reinfection. A documented reinfection was defined as a positive SARS-CoV-2 PCR or antigen test that occurred 60 or more days after a COVID-19 infection index date. Home COVID-19 tests administered outside a healthcare setting were not included in the dataset.

The subgroup of reinfected patients tended to be younger and more likely to have documented race and ethnicity information. Fewer reinfected patients (14.3%) had a documented vaccination prior to their COVID index date than patients who did not have reinfection (25.0%), a statistically significant finding (*p* < 0.0001) with a small effect size (Cramer’s *V* = 0.06). Although most reinfected individuals (*N* = 203,735) had only one reinfection, a small group (*N* = 478) had three or more reinfections. This group included a larger proportion of individuals with higher CCI scores (3+) and a smaller proportion of individuals with a documented vaccination compared to groups with fewer reinfections (Supplementary Data 1). Supplementary Data 1 provides a disaggregation of Table 1 by the number of reinfections.

**Fig. 1:**
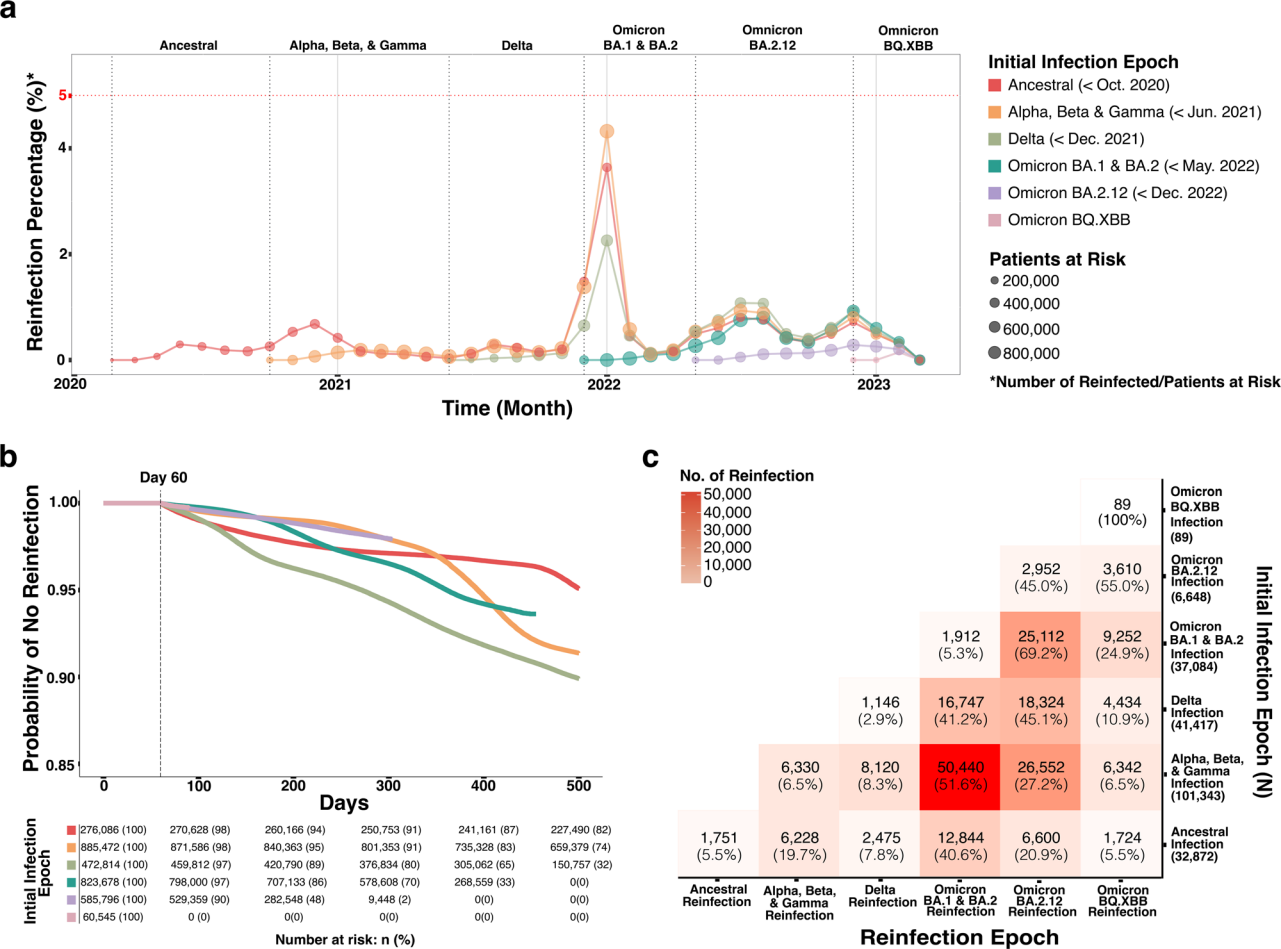
Timing and incidence of COVID-19 reinfection by variant. **a** Percentage of patients at risk that had a reinfection in each month. Distinct colors are used to indicate the epoch of initial infection and the size of the dot illustrates the number of persons with a reinfection. **b** Kaplan–Meier curve that shows time to event between initial COVID-19 infection and first COVID-19 reinfection by COVID-19 variant. Distinct colors represent distinct epochs of initial infection. **c** Heatmap of the relationship between the initial infection epoch and first reinfection epoch with darker red indicating a larger column percentage.

Figure 1 illustrates three approaches for understanding the occurrence of reinfection as it relates to COVID-19 variants. Figure 1a shows the percentage of patients at risk that had a reinfection each month. Distinct colors are used to indicate the epoch of initial infection and the size of the dot illustrates the number of persons with a reinfection. This figure is useful for conveying the varying likelihood of reinfection while accounting for individuals who passed away following their first infection since these individuals are no longer considered at risk. The initial epoch reflects the dominant COVID strain as identified by the CDC for specific time periods^23^. Figure 1a shows the largest increase in reinfections in the Omicron BA.1 and BA.2 epoch among individuals with initial infections during the Ancestral and Alpha, Beta, and Gamma periods, and a smaller increase among those first infected in the Delta epoch. The difference between these variants is smaller for reinfections in the Omicron BA.2.12 and Omicron BQ XBB epochs. Individuals with initial infections in the Delta variant have the largest reinfection percentage relative to other variants in the Omicron BA 2 A.2.12 period. The reinfection spikes appear to occur in winter months close to the holiday season and in the early summer.

Figure 1b is a Kaplan–Meier curve that shows the time to the event between the initial COVID-19 infection and the first COVID-19 reinfection by the COVID-19 variant. This figure is useful for understanding the days to infection, including demonstrating that in many cases, reinfection occurred more than 100 days after the initial COVID-19 infection. Some reinfections were much later: 156 individuals had a first documented reinfection more than 1000 days after initial COVID-19 infection. Figure 1b also shows that this analysis is not particularly sensitive to the decision to use a 60-day threshold rather than a 90-day threshold because few reinfections (*n* = 11,497, 5.4%) occur in the 60- to 90-day window for any variant. We recognize that this figure cannot appropriately account for individuals who passed away following their first infection. Due to the violation of proportional hazards evident in Fig. 1b, we have chosen to not report odds ratios because they may be misinterpreted. Figure 1c most clearly details the relationship between the variant of initial infection and the variant of reinfection, highlighting that reinfections in the Omicron BA 1 and 2 time period were particularly common among individuals initially infected in the Ancestral COVID-19 (40.6%) and Alpha, Beta, and Gamma (51.6%) epochs. For individuals with initial infections in the Delta epoch, the largest numbers of reinfections were during the Omicron BA 2.12 time period (45.1%)

**Fig. 2:**
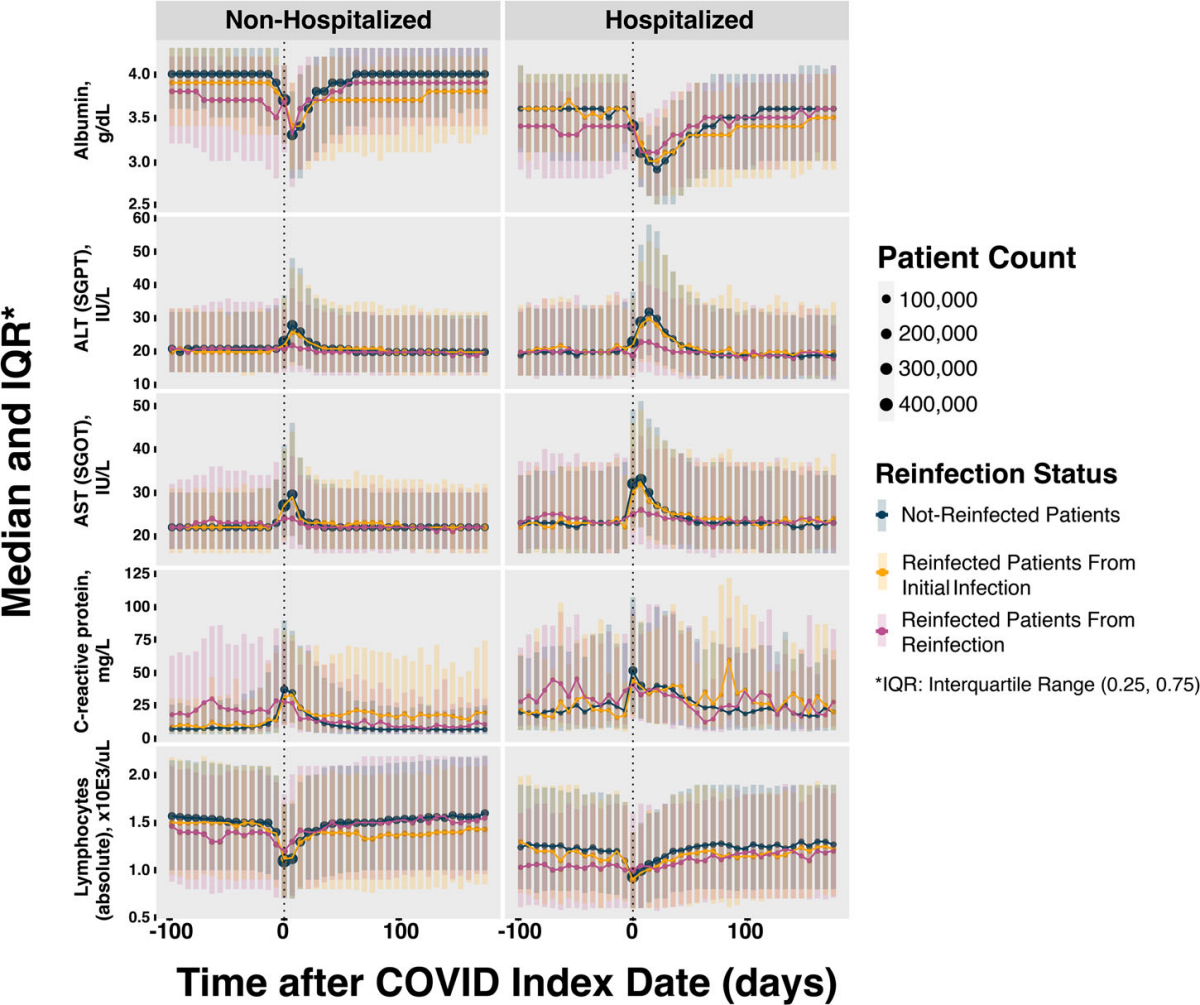
Biomarker trends of patients with and without reinfection disaggregated by hospitalization status for associated infection index date with interquartile range. Median and interquartile range for five biomarkers. Dark blue indicates results from non-reinfected patients, yellow indicates results from reinfected patients around their initial infection, and pink indicates results from reinfected patients around their reinfection.

We evaluated biomarker trends in Fig. 2, comparing the median laboratory values of patients with and without at least one reinfection. Comparisons were made between the index date of the initial infection and the subsequent first reinfection date. Biomarkers of hepatic inflammation (ALT and AST) were less elevated during acute reinfection compared to initial COVID-19 infection (ALT non-hospitalized-W: 1,984,798, *p*-value < 0.00; ALT hospitalized-W: 2,720,223, *p*-value < 0.001; AST non-hospitalized-W: 1,944,908, *p*-value < 0.001; AST non-hospitalized-W: 2,665,488, *p*-value < 0.001) and normalized over a similar time period. However, albumin trends show that among patients with reinfection, albumin levels were persistently lower after initial COVID-19 infection (non-hospitalized-W: 7,022,855, *p*-value < 0.001; hospitalized-W: 412,724, *p*-value < 0.001) and that levels were also lower prior to the reinfection date (non-hospitalized-W: 9,728,318, *p*-value < 0.001; hospitalized-W: 703,716, *p*-value < 0.001). We completed two sensitivity analyses for biomarkers with stricter COVID-19 indicator requirements: the first with a cohort that required a positive PCR or antigen test and the second with a cohort that required an ICD-10 diagnosis code. We found no notable difference in results with these cohorts.

### Severity of reinfection

We explored the characteristics of severity of infection and hospitalization in Table 2. The “No Documented Reinfection” column highlights that most individuals, even those with ED visits or hospitalization at the first documented infection, do not have documentation of a reinfection. The shaded portion of Table 2 summarizes results from individuals from their first documented reinfection. We performed a chi-square test of independence for both the entirety of Table 2 (with “No Reinfection” included) and for the shaded portion of Table 2. For the entire table, we found a statistically significant difference (chi-squared value: 28,690, *p* < 0.0001), although with a negligible effect size (Cramer’s *V*: 0.05, DoF = 4). For the shaded portion of the table where all individuals had a reinfection, we found a statistically significant difference (chi-squared value: 25,697, *p*-value: <0.0001) with a medium effect size (Cramer’s *V*: 0.20, DoF = 4). These results, particularly those among individuals who experience reinfection, suggest that the severity of reinfection may not be independent of the severity of the initial infection.

**Table 2:** Comparison of severity of first and reinfection with SARS-CoV-2

Count row (R)% among those with a reinfection^a^ column (C)%	Severity of reinfection						Total
Severity of initial infection	No documented reinfection	Mild with no ED visit or hospitalization around reinfection index	Mild with ED visit around reinfection index	Moderate with hospitalization around reinfection index	Severe with ECMO or IMV or vasopressor during hospitalization around reinfection index	Death within 60 days of reinfection index	
Mild with no ED visit or hospitalization around COVID Iindex	2,528,697 C: 87%	**161,132** **R: 87.4%**	**13,022** **R: 7.1%**	**7893** **R: 4.3%**	**931** **R: 0.5%**	**1383** **R: 0.8%**	2,713,058 (184,361 multiple infections) C: 87%
Mild with ED visit around COVID index	191,910 C: 6.6%	**9474** **R: 58.9%**	**5333** **R: 33.1%**	**1064** **R: 6.6%**	**127** **R: 0.8%**	**100** **R: 0.6%**	208,008 (16,098 multiple infections) C: 6.7%
Moderate with hospitalization around COVID index	145,160 5.0%	**6045** **R: 52.9%**	**1849** **R: 16.2%**	**2833** **R: 24.8%**	**277** **R: 2.4%**	**416** **R: 3.6%**	156,580 (11,420 multiple infections) C: 5.0%
Severe with ECMO or IMV or vasopressor during hospitalization around COVID Iindex	15,491 C: 0.5%	**501** **R: 45.3%**	**177** **R: 16.0%**	**268** **R: 24.3%**	**92** **R: 8.3%**	**67** **R: 6.1%**	16,596 (1105 multiple infections) C: 0.6%
Death within 60 days of COVID index	10,149 C: 0.4%	N/A	N/A	N/A	N/A	N/A	10,149 C: 0.3%
Total	2,891,407	177,152	20,381	12,058	1,427	1,966	3,104,391

^a^The items in bold indicate the values used for the calculation of row percentages among those with a SARS-CoV-2 reinfection. Individuals without a reinfection are not included in this calculation.

We completed two sensitivity analyses related to severity. First, we did an analysis requiring a PCR or antigen test for initial COVID-19 index date or a test within 7 days of ICD-10 diagnosis if ICD-10 diagnosis code was used as the index date (entire table: chi-squared = 34,622, *p* < 0.0001, Cramer’s *V* = 0.06, *p* < 0.0001; shaded portion of table: chi-squared value = 347,978, *p* < 0.0001, Cramer’s *V* = 0.26, DoF = 4). Then, we did an analysis requiring an ICD-10 diagnosis code as initial COVID index date or use of an ICD-10 diagnosis code within 7 days of a PCR or antigen test if a test was used as the index date (entire table: chi-squared = 18,329, *p* < 0.0001, Cramer’s *V* = 0.05, shaded portion of table: chi-squared = 13,017, *p* < 0.0001, Cramer’s *V* = 0.20, DoF = 4). In both cases, we validated the existing findings.

Overall, most individuals in the cohort (87.5%) had a mild initial infection without an ED visit. Of those with a mild initial infection without an ED visit, the large majority (87.4%) of first reinfections did not coincide with an ED visit or hospitalization. Among those with an ED visit during their first infection, approximately a third (33.1%) of first reinfections included an ED visit. Of those hospitalized at the first infection, more than a quarter (27.2%) of first reinfections required hospitalization. Fewer than half (45.3%) of first reinfections among individuals who experienced a severe initial infection were mild. 8.3% were severe, and 6.1% occurred within 60 days of death. Nearly a quarter (24.3%) of first reinfections among individuals with a severe initial infection were moderate.

The average age at the COVID index of individuals with both a mild initial infection and a mild first reinfection not coinciding with an ED visit was 44.2, and the average CCI score was 0.9 (*n* = 161,132). The average age at the COVID index of individuals with both a severe initial infection and a severe first reinfection was 60.2, and the average CCI score was 5.6 (*n* = 92). The differences in mean age and CCI score are both statistically significant (null hypothesis: *μ*1−*μ*2 = 0; alpha = 0.05; *p* < 0.0001 for both age and CCI).

Approximately 79% of individuals who were not hospitalized for either their initial infection or first reinfection had a Charlson comorbidity index (CCI) score of 4 or >3, while 32,134.8% of individuals who were hospitalized for their initial infection and 47,748.8% of individuals who were hospitalized for their first reinfection had a CCI score larger than 3 of 4 or greater. These differences were statistically significant (null hypothesis: *μ*1–*μ*2 = 0; alpha = 0.05, *p* < 0.0001).

We provide additional details in Supplementary Data 2 with a breakdown of demographic details for patients who experience hospitalization for COVID-19. Individuals who were hospitalized during reinfection have similar race and ethnicity characteristics to those hospitalized during the initial infection, though statistically significant differences (*p* < 0.0001) remain between those who were and were not hospitalized. In Supplementary Data 3, we provide Table 2 disaggregated by age, noting that disclosure requirements may limit the robustness of conclusions.

### Reinfection and diagnosis of Long COVID

**Fig. 3:**
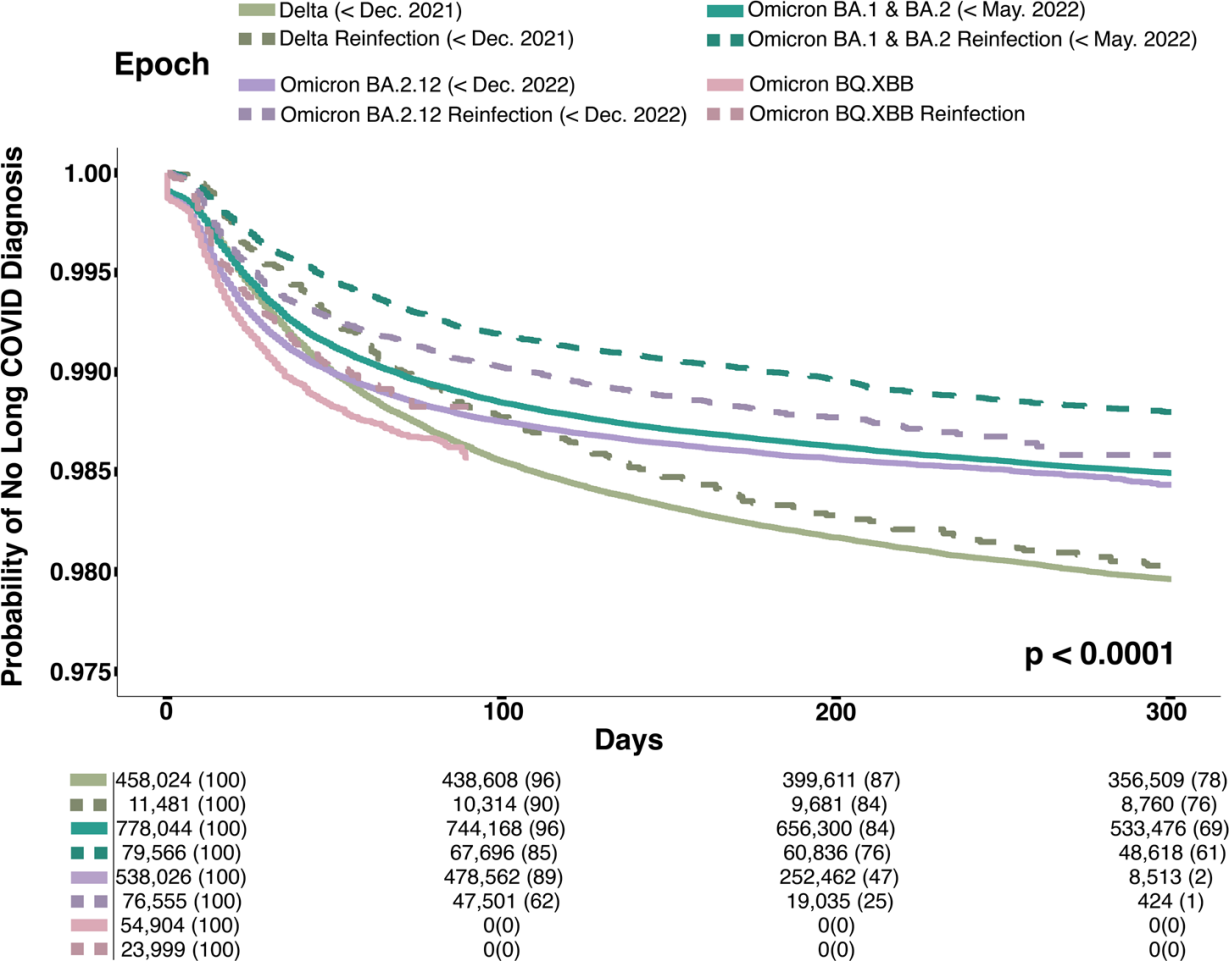
Kaplan–Meier curves for time to Long COVID diagnosis after initial infection versus after first reinfection disaggregated by variant. Kaplan–Meier curve illustrating the time to Long COVID diagnosis following initial infection or reinfection. Results are disaggregated by infection epoch, with the Delta variant in green, the Omicron variant in blue, and the Omicron BA variant in purple. For each color, the lighter shade represents the initial infection, and the darker shade represents reinfection.

Finally, Fig. 3 shows the Kaplan–Meier curves for time to Long COVID diagnosis after the initial infection versus after the first reinfection. The findings suggest a statistically significant difference in trajectories (*p* < 0.001). For all variant epochs, the incidence of new Long COVID diagnoses after reinfection is lower than after initial COVID-19 infection. The rate of Long COVID diagnoses following the initial infection and first reinfection was largest in the Delta epoch and smallest in the Omicron BA 1 and 2 epoch. The time for follow-up is the shortest during the Omicron BQ.XBB epoch.

Two sensitivity analyses were conducted to assess the robustness of the findings related to Long COVID, the first with a cohort that used a positive PCR or antigen test for the COVID-19 index date and the second with a cohort that used an ICD-10 diagnosis code for the COVID-19 index date (not shown). Individuals with an ICD-10 diagnosis code for the COVID-19 index date were more likely to be diagnosed with Long COVID and more likely to be diagnosed sooner than individuals with a positive PCR or antigen test for the COVID-19 index date. However, alternative explanations exist for this finding, such as that the cohort with only a COVID-19 ICD-10 diagnosis for index date was older and had more comorbidities than the cohort with a PCR or AG test for COVID-19 index date (Supplementary Data 3).

## Discussion

The overall proportion of individuals with at least one documented reinfection in the cohort (6.9%) was larger than the upper bound of incidence of reinfection noted in the literature (5.5%)^5^. This is likely an underestimate given the advent of home testing, particularly in more recent epochs when home testing has been even more accessible. Most individuals in the cohort had a PCR or antigen test around the time of initial infection. Similar to existing findings, the large majority of reinfections occurred during the Omicron epoch. Other studies have suggested that the considerable increase in COVID infections and reinfections during Omicron may be a result of waning immunity, high transmissibility, and immune escape^36–38^. Figure 1 suggests that the increase in reinfections during Omicron BA.1 & BA.2 holds regardless of the epoch of the initial COVID-19 infection. The large number of reinfections during Omicron BA.1 and BA.2 makes it challenging to draw conclusions about comparing reinfections between variants because there may be other factors, such as adherence to masking or social distance policies, that impacted the likelihood of exposure and subsequent reinfections. However, it is notable that in Fig. 1, initial infection during Delta appeared to be more protective against reinfection during Omicron BA.1 & BA.2 than initial infection during Ancestral COVID or Alpha, Beta, and Gamma. This difference disappeared during Omicron BA 2.12, though this may be explained by a lack of follow-up time rather than a variant. Previous studies have documented reinfections a median of 269–411 days after initial COVID-19 infection^9^. This study finds evidence of reinfections that occur more than 300 days and up to nearly 1100 days after initial COVID-19 infection. This work also validates the occurrence of multiple reinfections, including a small subset of individuals with three or more reinfections.

The mean age of reinfected individuals is close to five years lower than the mean age of those without reinfection. This aligns with literature that suggests that reinfections are more commonly reported for younger individuals^10^. One study found that the reinfection rate was highest among those aged 18–29 years old^39^ while public health data collected from September 2021 through September 2022 in the state of Washington found disproportionately large numbers of individuals with reinfections in the 18- to 34- and 35- to 49-year old age groups^40^. One possible explanation is that younger age groups are less likely to use COVID-19 preventative measures like social distancing and more likely to engage with others for work and leisure, leading to multiple exposures and reinfections^41^. Another possible explanation is that younger individuals are less likely to be vaccinated and may be more susceptible to reinfection^42^. A third possible explanation is that older individuals were more likely to die following the initial infection, so more reinfections occurred among younger individuals^43^. A fourth possible explanation is that young adults have high rates of asymptomatic and paucisymptomatic infection, which may be less protective of reinfection^44^. More research is needed to explore these potential explanations.

Women make up nearly two-thirds of the study cohort and a larger proportion of those with a documented reinfection. Some studies have also noted larger proportions of women than men with reinfections and suggested that there may be relevant differences in immune response by sex^45–48^. However, Supplemental Fig. 1 suggests that the inclusion criteria requiring at least two visits in the year prior to the COVID-19 index date contribute to this imbalance. Previous research also suggests that women are more likely to utilize health services and demonstrate health-seeking behavior^49,50^. The sex imbalance in these findings may be more likely associated with differential healthcare utilization rather than biological differences.

Individuals with one or more reinfections are more likely to have a higher CCI score and less likely to be vaccinated than individuals without a documented reinfection. These findings are in alignment with literature that suggests that vaccination can have a protective effect against reinfection and that comorbidities may be associated with reinfection^11,51,52^. However, the overall rate of vaccination in this study is low as this study includes substantial time when vaccinations were not available and does not account for variation in vaccination reporting by site. Further research could account for vaccination data quality concerns, timing, and type of vaccination as related to protection against reinfection with particular variants, as well as a more complex analysis of the relationship between CCI scores and reinfection.

Biomarkers have been well studied in acute COVID-19 infection, and several laboratory markers have been associated with higher severity of infection and mortality^53,54^. Our work extends knowledge of biomarkers to reinfections. In finding that biomarkers of hepatic inflammation were less elevated during acute reinfection compared to acute initial infection, we contribute a novel result that has not yet been reported to the best of our knowledge. Another new finding to the best of our knowledge, is that albumin appears to be lower leading up to reinfection. Previous studies have suggested that hypoalbuminemia is common in COVID-19 patients, and dynamic monitoring of serum albumin may be useful in evaluating the risk of reinfection with COVID-19^55–58^. Albumin has also been shown to be among the biomarkers associated with long COVID symptoms^18^. We suggest that further work may explore if lower albumin levels may be a predictor of COVID-19 reinfection.

Similar to previous studies measuring the severity of reinfection through hospitalization, we find that most individuals did not have an ED visit or hospitalization at the time of either first infection or reinfection. We contribute novel findings to the best of our knowledge that individuals who were hospitalized at the time of the initial infection are potentially at much greater risk for hospitalization during reinfection. The effect size is medium when we consider the results among those with reinfections, noting that we cannot account for what would have happened among those who passed away following their first infection. Our sensitivity analysis validates the robustness of our findings when considering a cohort requiring PCR or antigen test for initial COVID index separately from a cohort requiring COVID-19 ICD-10 diagnosis code.

We first consider the varying degrees of severity of initial infection among individuals who also have a reinfection. Approximately a third (33.1%) of individuals with a mild initial infection that coincided with an ED visit also had an ED visit that coincided with the time of their reinfection. This proportion is larger than the respective proportion of individuals who had a moderate or severe hospitalization during the first infection and an ED visit following reinfection. Further analysis could investigate if the group of individuals who visited an ED for both first infection and reinfection reflects ED-seeking behavior that may result from the convenience of the ED, limited access to other healthcare options, or health insurance status^59^.

More than a quarter of individuals with either a moderate or severe first infection coinciding with hospitalization also were hospitalized at the time of reinfection. Although this is concerning, a promising finding is that the proportion experiencing severe reinfection coinciding with the use of extracorporeal membrane oxygenation (ECMO), invasive mechanical ventilation (IMV), or vasopressors during hospitalization was small (2.4% of those with moderate first infection and 8.3% of those with severe first infection). One possible interpretation is that experiencing hospitalization during reinfection may be related to experiencing hospitalization during the first infection, but the hospitalization may be less severe. A second possible explanation is that later variants tend to cause less severe infection. A third possible explanation for the reduction in treatments during reinfection-associated hospitalizations is that clinical thresholds for this treatment or clinical behavior may have changed over time. We also observe that the proportion of individuals who pass away following reinfection is higher than the proportion who experience severe reinfection among those with an initial moderate infection and lower for those who have a severe initial infection. It is likely that some patients have higher rates of hospitalization in general due to other underlying conditions. This is supported by our that the small group of individuals who were hospitalized and required ECMO and IMV for both initial and reinfection is older and has a higher average CCI score than individuals who experienced mild first and reinfections that did not coincide with hospitalization. We suggest further analysis to better understand attributes that are predictive of moderate and severe reinfections.

This study contributes novel findings to the best of our knowledge of the relationship between reinfection with Long COVID diagnosis. The largest proportion of Long COVID diagnoses occur among individuals with a first reinfection in the Delta epoch. The rate of Long COVID diagnoses has been increasing with each successive Omicron variant, which is particularly notable as there has been less follow-up time for variants such as Omicron BQ.XBB. Several possible explanations exist for these associative findings. One is that there may be a biological explanation where reinfection may be associated with an increased risk of post-acute sequelae. This has been suggested in other literature^25^. Another explanation is that physician diagnosing behavior has changed, and physicians are more likely to have adopted the use of either the new U09.9 Long COVID diagnosis code or the existing B94.8 code in more recent variants. This work has also not accounted for other factors like the impact of vaccination status or the use of outpatient therapeutics like Paxlovid in relation to Long COVID. Future analysis could explore a causal relationship between reinfection and Long COVID and account for these other factors.

A limitation of this study is the reliance on EHR data. EHR limitations are well documented and include selection bias based on varying rates of healthcare utilization, concerns about fitness for purpose and drawing inappropriate conclusions, and data quality and missing data challenges^60^. EHR studies also differ from clinical studies, where patients may be followed more closely. A major limitation of this particular analysis is that we are limited to EHR collected at specific hospitals; we cannot join patient records between hospitals. Strategies we have used to address these limitations include hospitalization inclusion criteria for sites and visit inclusion criteria for individuals that promote more detailed, robust, and higher-quality data.

A second limitation is that it is not feasible to include the results of home COVID-19 tests. Individuals are likely testing positive for COVID-19 reinfections that are not documented in this dataset. This may result in an underestimate of the number of individuals with reinfections. The varying availability of home tests over the duration considered for this project may result in an uneven impact of this limitation. To address this limitation, we have attempted to maintain a focus on behaviors that require healthcare interaction, such as Long COVID diagnosis, biomarkers, and hospitalization. We do not suggest the generalizability of results to situations that would not involve healthcare settings. We also limited analysis to individuals who had an initial COVID infection prior to December 31, 2022, in an effort to focus on a window in time when home tests were less common. Future work could explore which time periods best capture testing for reinfection.

A third limitation is that we limit the analyses of biomarkers, severity, and Long COVID diagnosis to only the first COVID-19 reinfection. This is due to the small number of individuals with multiple reinfections. Subsequent analyses could further explore the impact of multiple reinfections.

A fourth limitation is that the use of inclusion criteria permitting only a COVID-19 diagnosis (rather than requiring a PCR or antigen test) may result in an inaccurate COVID-19 initial index date if the diagnosis is not well aligned with an infection. This inaccuracy could impact the identification of reinfections. The impacted cohort is a smaller portion (27%) of our dataset and sensitivity analyses for biomarkers and severity suggest that the results are robust to inclusion of this group. We continue to include individuals with only a diagnosis for the first infection as there may be other information (such as a home test) that was used to make the diagnosis that is not available to us.

A fifth limitation is that the definition of reinfection only considers PCR or antigen tests after the COVID index date and does not consider COVID-19 ICD-10 diagnoses that occur in the EHR after the index date. We explored the inclusion of the COVID-19 ICD-10 diagnosis codes as reinfections but observed a dramatic and likely unrealistic increase in the number of patients with 5 or more supposed reinfections using this expanded definition. With available data, it was impossible to determine which new uses of a COVID-19 ICD-10 diagnosis code at a visit to the EHR reflected a new reinfection or new COVID symptoms as opposed to documentation of a historical experience that occurred at some unknown time prior to the visit. Authors chose to prioritize reinfections confirmed through testing with the acknowledgement that this decision likely undercounts reinfection. Future work using advances in modeling and natural language processing could help to better distinguish when a COVID ICD-10 diagnosis code alone signals a reinfection.

Reinfections are well documented in an EHR-based cohort from the RECOVER initiative and align with overall incidence rates in the literature. This work validates existing characterization of reinfection as most common in the Omicron epoch and contributes a novel characterization to the best of our knowledge of lower albumin levels after initial COVID-19 infection and leading up to reinfection. The severity of reinfection appears to be associated with the severity of initial infection, and Long COVID diagnoses appear to occur more often following initial infection than reinfection in the same epoch. We describe several opportunities for further research with these findings to better understand COVID-19 reinfections.

### Supplementary information

Supplementary Information

Description of Additional Supplementary Files

Supplementary Data 1

Supplementary Data 2

Supplementary Data 3

Supplementary Data 4

Supplementary Data 5

Reporting Summary

## Data Availability

All data used in this study is available through the N3C Enclave to approved users. See https://covid.cd2h.org/for-researchers for instructions on how to access the data. We used N3C data from version 141 (9/14/2023). Source data for figures is available in Supplementary Data 5.

## Code availability

All analysis and visualization were done in the N3C Enclave using SQL, Python (v3.6), and R (v3.6), including ggplot2, survival, and survminer packages. Code is available on Zenodo^61^.
