# Supplementary material for: Insights from an N3C RECOVER EHR-based cohort study characterizing SARS-CoV-2 reinfections and Long COVID
Source: Commun Med (Lond). 2024 Jul 11;4:129. doi: 10.1038/s43856-024-00539-2 (PMC11239932; doi:10.1038/s43856-024-00539-2)
Supplement: Supplementary file 1 — Supplementary Information [file 43856_2024_539_MOESM1_ESM.pdf]

Supplementary Information

Supplementary Figure 1. Data filtering process with sex ratio.

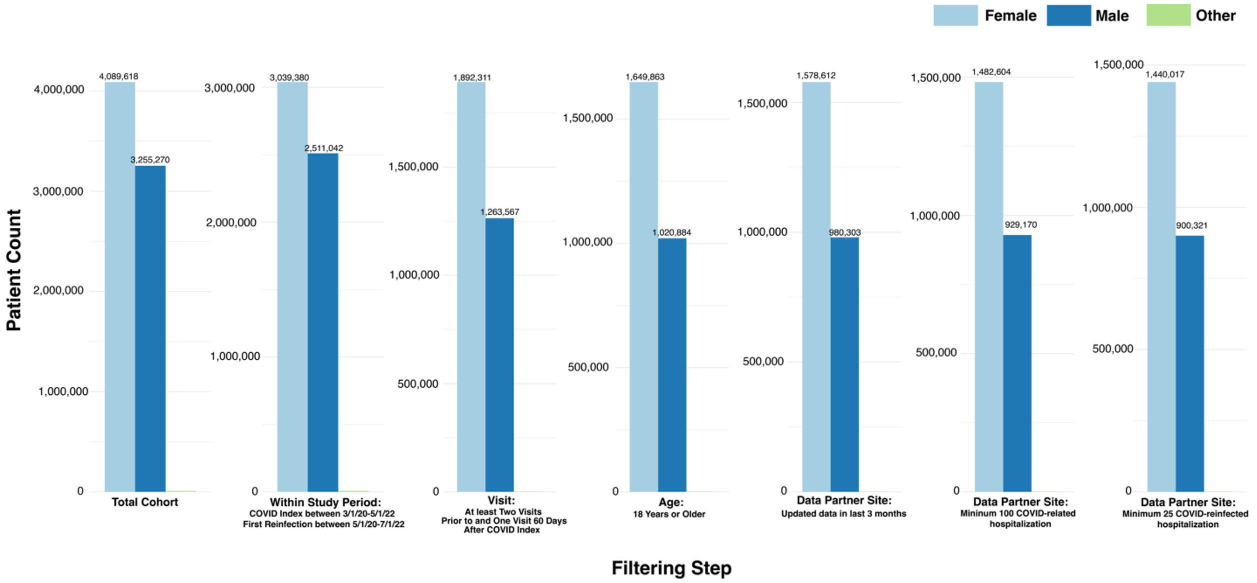

Supplementary Figure 2: Count of individuals by measurement used for initial COVID-19 index date.

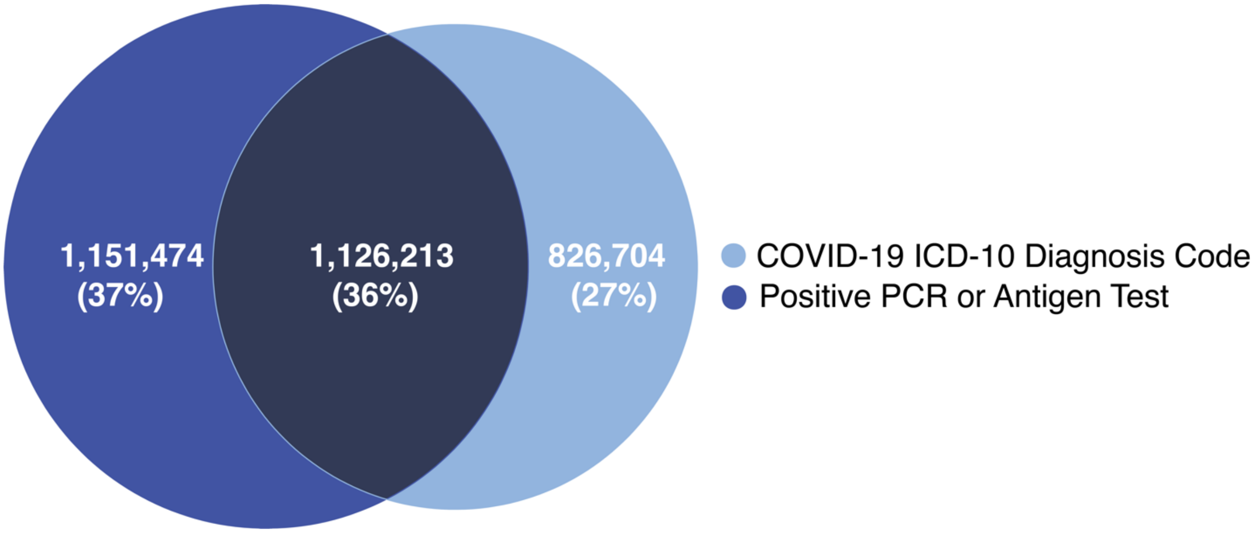

Supplementary Acknowledgements: We are grateful to the following data partners for their many contributions.

Data Partners with Released Data
Institutions whose data are released or pending:
Available: Advocate Health Care Network — UL1TR002389: The Institute for Translational Medicine (ITM) • Boston University Medical Campus — UL1TR001430: Boston University

Clinical and Translational Science Institute • Brown University — U54GM115677: Advance Clinical Translational Research (Advance-CTR) • Carilion Clinic — UL1TR003015: iTHRIV Integrated Translational health Research Institute of Virginia • Charleston Area Medical Center — U54GM104942: West Virginia Clinical and Translational Science Institute (WVCTSI) • Children’s Hospital Colorado — UL1TR002535: Colorado Clinical and Translational Sciences Institute • Columbia University Irving Medical Center — UL1TR001873: Irving Institute for Clinical and Translational Research • Duke University — UL1TR002553: Duke Clinical and Translational Science Institute • George Washington Children’s Research Institute — UL1TR001876: Clinical and Translational Science Institute at Children’s National (CTSA-CN) • George Washington University — UL1TR001876: Clinical and Translational Science Institute at Children’s National (CTSA-CN) • Indiana University School of Medicine — UL1TR002529: Indiana Clinical and Translational Science Institute • Johns Hopkins University — UL1TR003098: Johns Hopkins Institute for Clinical and Translational Research • Loyola Medicine — Loyola University Medical Center • Loyola University Medical Center — UL1TR002389: The Institute for Translational Medicine (ITM) • Maine Medical Center — U54GM115516: Northern New England Clinical & Translational Research (NNE-CTR) Network • Massachusetts General Brigham — UL1TR002541: Harvard Catalyst • Mayo Clinic Rochester — UL1TR002377: Mayo Clinic Center for Clinical and Translational Science (CCaTS) • Medical University of South Carolina — UL1TR001450: South Carolina Clinical & Translational Research Institute (SCTR) • Montefiore Medical Center — UL1TR002556: Institute for Clinical and Translational Research at Einstein and Montefiore • Nemours — U54GM104941: Delaware CTR ACCEL Program • NorthShore University HealthSystem — UL1TR002389: The Institute for Translational Medicine (ITM) • Northwestern University at Chicago — UL1TR001422: Northwestern University Clinical and Translational Science Institute (NUCATS) • OCHIN — INV-018455: Bill and Melinda Gates Foundation grant to Sage Bionetworks • Oregon Health & Science University — UL1TR002369: Oregon Clinical and Translational Research Institute • Penn State Health Milton S. Hershey Medical Center — UL1TR002014: Penn State Clinical and Translational Science Institute • Rush University Medical Center — UL1TR002389: The Institute for Translational Medicine (ITM) • Rutgers, The State University of New Jersey — UL1TR003017: New Jersey Alliance for Clinical and Translational Science • Stony Brook University — U24TR002306 • The Ohio State University — UL1TR002733: Center for Clinical and Translational Science • The State University of New York at Buffalo — UL1TR001412: Clinical and Translational Science Institute • The University of Chicago — UL1TR002389: The Institute for Translational Medicine (ITM) • The University of Iowa — UL1TR002537: Institute for Clinical and Translational Science • The University of Miami Leonard M. Miller School of Medicine — UL1TR002736: University of Miami Clinical and Translational Science Institute • The University of Michigan at Ann Arbor — UL1TR002240: Michigan Institute for Clinical and Health Research • The University of Texas Health Science Center at Houston — UL1TR003167: Center for Clinical and Translational Sciences (CCTS) • The University of Texas Medical Branch at Galveston — UL1TR001439: The Institute for Translational Sciences • The University of Utah — UL1TR002538: Uhealth Center for Clinical and Translational Science • Tufts Medical Center — UL1TR002544: Tufts Clinical and Translational Science Institute • Tulane University — UL1TR003096: Center for Clinical and Translational Science • University Medical Center New Orleans — U54GM104940: Louisiana Clinical and Translational Science (LA CaTS) Center • University of Alabama at Birmingham — UL1TR003096: Center for Clinical and Translational Science • University of Arkansas for Medical Sciences — UL1TR003107: UAMS Translational Research Institute • University of Cincinnati — UL1TR001425: Center for Clinical and Translational Science and Training • University of Colorado Denver, Anschutz Medical Campus — UL1TR002535: Colorado Clinical and Translational Sciences Institute • University of Illinois at Chicago — UL1TR002003: UIC Center for Clinical and Translational Science • University of Kansas Medical Center — UL1TR002366: Frontiers: University of

Kansas Clinical and Translational Science Institute • University of Kentucky — UL1TR001998: UK Center for Clinical and Translational Science • University of Massachusetts Medical School Worcester — UL1TR001453: The UMass Center for Clinical and Translational Science (UMCCTS) • University of Minnesota — UL1TR002494: Clinical and Translational Science Institute • University of Mississippi Medical Center — U54GM115428: Mississippi Center for Clinical and Translational Research (CCTR) • University of Nebraska Medical Center — U54GM115458: Great Plains IDeA-Clinical & Translational Research • University of North Carolina at Chapel Hill — UL1TR002489: North Carolina Translational and Clinical Science Institute • University of Oklahoma Health Sciences Center — U54GM104938: Oklahoma Clinical and Translational Science Institute (OCTSI) • University of Rochester — UL1TR002001: UR Clinical & Translational Science Institute • University of Southern California — UL1TR001855: The Southern California Clinical and Translational Science Institute (SC CTSI) • University of Vermont — U54GM115516: Northern New England Clinical & Translational Research (NNE-CTR) Network • University of Virginia — UL1TR003015: iTHRIV Integrated Translational health Research Institute of Virginia • University of Washington — UL1TR002319: Institute of Translational Health Sciences • University of Wisconsin-Madison — UL1TR002373: UW Institute for Clinical and Translational Research • Vanderbilt University Medical Center — UL1TR002243: Vanderbilt Institute for Clinical and Translational Research • Virginia Commonwealth University — UL1TR002649: C. Kenneth and Dianne Wright Center for Clinical and Translational Research • Wake Forest University Health Sciences — UL1TR001420: Wake Forest Clinical and Translational Science Institute • Washington University in St. Louis — UL1TR002345: Institute of Clinical and Translational Sciences • Weill Medical College of Cornell University — UL1TR002384: Weill Cornell Medicine Clinical and Translational Science Center • West Virginia University — U54GM104942: West Virginia Clinical and Translational Science Institute (WVCTSI)

Submitted:
Icahn School of Medicine at Mount Sinai — UL1TR001433: ConduITS Institute for Translational Sciences • The University of Texas Health Science Center at Tyler — UL1TR003167: Center for Clinical and Translational Sciences (CCTS) • University of California, Davis — UL1TR001860: UCDavis Health Clinical and Translational Science Center • University of California, Irvine — UL1TR001414: The UC Irvine Institute for Clinical and Translational Science (ICTS) • University of California, Los Angeles — UL1TR001881: UCLA Clinical Translational Science Institute • University of California, San Diego — UL1TR001442: Altman Clinical and Translational Research Institute • University of California, San Francisco — UL1TR001872: UCSF Clinical and Translational Science Institute
Pending: Arkansas Children's Hospital — UL1TR003107: UAMS Translational Research Institute • Baylor College of Medicine — None (Voluntary) • Children's Hospital of Philadelphia — UL1TR001878: Institute for Translational Medicine and Therapeutics • Cincinnati Children's Hospital Medical Center — UL1TR001425: Center for Clinical and Translational Science and Training • Emory University — UL1TR002378: Georgia Clinical and Translational Science Alliance • HonorHealth — None (Voluntary) • Loyola University Chicago — UL1TR002389: The Institute for Translational Medicine (ITM) • Medical College of Wisconsin — UL1TR001436: Clinical and Translational Science Institute of Southeast Wisconsin • MedStar Health Research Institute — UL1TR001409: The Georgetown-Howard Universities Center for Clinical and Translational Science (GHUCCTS) • MetroHealth — None (Voluntary) • Montana State University — U54GM115371: American Indian/Alaska Native CTR • NYU Langone Medical Center — UL1TR001445: Langone Health's Clinical and Translational Science Institute • Ochsner Medical Center — U54GM104940: Louisiana Clinical and Translational Science (LA CaTS) Center • Regenstrief Institute — UL1TR002529: Indiana Clinical and Translational Science Institute • Sanford Research — None (Voluntary) • Stanford University —

UL1TR003142: Spectrum: The Stanford Center for Clinical and Translational Research and Education • The Rockefeller University — UL1TR001866: Center for Clinical and Translational Science • The Scripps Research Institute — UL1TR002550: Scripps Research Translational Institute • University of Florida — UL1TR001427: UF Clinical and Translational Science Institute • University of New Mexico Health Sciences Center — UL1TR001449: University of New Mexico Clinical and Translational Science Center • University of Texas Health Science Center at San Antonio — UL1TR002645: Institute for Integration of Medicine and Science • Yale New Haven Hospital — UL1TR001863: Yale Center for Clinical Investigation

Supplementary Consortia: National COVID Cohort Collaborative (N3C) Consortia Authors
Axle Informatics, North Bethesda, MD, USA
Kristen Hansen

Berkeley Lab, Berkley, CA, USA
Justin Reese

Columbia University, New York, NY, USA
Karthik Natarajan

Datavant, San Francisco, CA, USA,
Jasmin Phua

Duke University, Durham, NC, USA
Warren Kibbe, PI

Emory University, Atlanta, GA, USA
Richard Moffitt, PI
Margaret Hall
Rishi Kamaleswaran
Jason Yoo

Endeavor Health, Evanston, IL, USA
Anthony Solomonides

Illinois State University, Normal, IL, USA
Nariman Ammar

Jackson Labs, Bar Harbor, ME, USA
Hannah Blau
Peter Robinson

Johns Hopkins University, Baltimore, MD, USA
Christopher G. Chute, PI
Ali Afshar
G. Caleb Alexander
Lisa Eskenazi
Tricia Francis
Davera Gabriel
Kirby Gong
Stephanie Hong

Harold Lehmann
Hemal Mehta
Chirag Parikh
Ann Parker
Rayna Xiao
Tanner Zhang
Richard Zhu
Jared Zook

Minderoo Center for Federated Cancer Research, Broadway Nedlands, WA, USA
Robert Miller

National Institutes of Health, Bethesda, MD, USA
Hythem Sidky

Northeastern University, Boston, MA, USA
Christian Reich, PI
Kristin Kostka

Oregon Community Health Information Network (OCHIN), Portland, OR, USA
Brenda McGrath, PI
Treasure Allen
Rob Schuff

Oregon Health & Science University, Portland, OR, USA
Erik Benton
David A. Dorr
Justin Ramsdill

Palantir Technologies, Denver, CO, USA
Maya Choudhury
Andrew Girvin

Patient Lead Research Collaborative, Calabasas, CA, USA
Hannah Davis, PI
Gina Assaf
Lisa McCorkell
Yochai Re'em
Anisha Sekar
Hannah Wei

RTI International, Durham, NC, USA
Daniel Brannock
Rob Chew
Emily Hadley
Alexander Preiss

Scripps, San Diego, CA, USA
Ginger Tsueng

State University of New York at Stony Brook, Stony Brook, NY, USA
Janos Hajagos, PI
Rachel Wong, PI
Adit Anand
Raj Gupta
Mengyao Hu
Prahathish Kameswaran
Saarthak Kapse
Eileen Keck
Farrukh koraishy
Spencer Krichevsky
Saaya Patel
Joel Saltz
Mary Saltz
Hiteshwar Singh
Shreya Sinha
Sam Soff
Kimon Stathakos
Chelsea Twan
Rohith Vaddavalli
Sai Rachana Yerram

The National Institute of Diabetes and Digestive and Kidney Diseases, Bethesda, MD, USA
Kenneth J. Wilkins

TriNetX, Cambridge, MA, USA
Lora Lingrey
Matvey Palchuk

Trinity Health, Livonia, MI, USA
Joe Flack

Tufts Medical Center, Boston, MA, USA
Andrew Williams, PI

University of California Davis, Davis, CA, USA
Konstantin Kunze, PI

University of Chicago, Chicago, IL, USA
Tom Best, PI

University of Colorado Anschutz Medical Campus, Aurora, CO, USA
Melissa Haendel, PI
Aleiya Anglo
Ian Brooks
Jonathan Dekermanjian
Peter DeWitt
Tursynay Issabekova
Michael Kahn

Bryan Laraway
Sruthi Magesh
Julie McMurry
Anh Nguyen
Jenny Nguyen
Shawn T. O'Neil
Meg Rebull
Chris Roeder
Seth Russell
L'tonya Starr
Anita Walden

University of Iowa, Iowa City, IA, USA
Dave Eichmann, PI
Charisse Madlock

University of Kansas Medical Center, Kansas City, KS, USA
Kelechi Anuforo, PI

University of Kentucky, Lexington, KY, USA
Ramakanth Kavuluru

University of Maryland Baltimore, Baltimore, MD, USA
Stacy Dalton, PI

University of Massachusetts, Amherst, MA, USA
Feifan Liu, PI

University of Michigan, Ann Arbor, MI, USA
J. Brian Byrd

University of Minnesota, Minneapolis, MN, USA
Steve Johnson, PI
Ashley Benner
Carolyn Bramante
Scott Chapman
Duy Duong
Michael Evans
Jared Huling
Corey McGee
Zheng Wang
Talia Wiggen
Rui Zhang

University of Nebraska Medical Center, Omaha, NE, USA
Jerrod Anzalone

University of North Carolina at Chapel Hill, Chapel Hill, NC, USA
Emily Pfaff, PI
Til Stürmer

Monika Baskaran
Abhishek Bhatia
Marshall Clark
Sofia Dard
Michele Jonsson Funk
Liz Kelly
Paul Kovach
Peter Leese
Tomas McIntee
JP Powers
Kellie Walters

University of Pittsburgh, Pittsburgh, PA, USA
Michele Morris

University of Rochester, Rochester, NY, USA
Elaine Hill, PI
Jack Chang
Adam Dziorny
Daniel Guth
Tanzy Love
Klint Mane
Sharad Kumar Singh
Richa Yadav
Ayushi

University of Texas Medical Branch, Galveston, TX, USA
Heidi Spratt, PI

University of Utah, Salt Lake City, UT, USA
Jackson Barlocker

University of Virginia, Charlottesville, VA, USA
Don Brown, PI
Sihang Jiang
Johanna Loomba
Saurav Sengupta
Suchetha Sharma
Andrea Zhou

University of Washington, Seattle, WA, USA
Rena Patel

University of Texas Health Science Center at Houston, Houston, TX, USA
Hongfang Liu, PI

Virginia Commonwealth University, Richmond, VA, USA
Brian Bush, PI
