# Supplementary material for: Insights from an N3C RECOVER EHR-based cohort study characterizing SARS-CoV-2 reinfections and Long COVID
Source: Commun Med (Lond). 2024 Jul 11;4:129. doi: 10.1038/s43856-024-00539-2 (PMC11239932; doi:10.1038/s43856-024-00539-2)
Supplement: Supplementary file 2 — Description of Additional Supplementary Files [file 43856_2024_539_MOESM2_ESM.pdf]

Description of Additional Supplementary Files.

File name: Supplementary Data 1

File Description: Demographic information in Table 1 disaggregated by number of COVID-19 infections.

File name: Supplementary Data 2

File Description: Demographic information in Table 1 disaggregated by COVID-19 hospitalization status.

File name: Supplementary Data 3

File Description: COVID-19 infection severity information in Table 2 disaggregated by age.

File name: Supplementary Data 4

File Description: Demographic information in Table 1 disaggregated by COVID-19 diagnosis type.

File name: Supplementary Data 5

File Description: Source data for figures in manuscript.
